# Peer Mobilization and Human Immunodeficiency Virus (HIV) Partner Notification Services Among Gay, Bisexual, and Other Men Who Have Sex With Men and Transgender Women in Coastal Kenya Identified a High Number of Undiagnosed HIV Infections

**DOI:** 10.1093/ofid/ofab219

**Published:** 2021-04-29

**Authors:** Maartje Dijkstra, Khamisi Mohamed, Alex Kigoro, Teresia Mumba, Shally Mahmoud, Abdalla Wesonga, Nana Mukuria, Tony Oduor, Evans Gichuru, Maarten F Schim van der Loeff, Shaun Palmer, Susan M Graham, Elise M van der Elst, Eduard J Sanders

**Affiliations:** 1 Department of Infectious Diseases, Public Health Service Amsterdam, Amsterdam, The Netherlands; 2 Division of Infectious Diseases, Department of Internal Medicine, Amsterdam Institute for Infection and Immunity, Amsterdam University Medical Center, Academic Medical Center, University of Amsterdam, The Netherlands; 3 Kenya Medical Research Institute–Wellcome Trust Research Program, Kilifi, Kenya; 4 University of Washington, Seattle, Washington, USA; 5 Nuffield Department of Medicine, University of Oxford, Oxford, United Kingdom; 6 Department of Global Health, Amsterdam University Medical Center, Academic Medical Center, University of Amsterdam, Amsterdam, The Netherlands

**Keywords:** acute HIV infection, partner notification, self-testing, sexual and gender minorities, sub-Saharan Africa

## Abstract

**Background:**

Human immunodeficiency virus (HIV) partner notification services (HPN), peer mobilization with HIV self-testing, and acute and early HIV infection (AEHI) screening among gay, bisexual, and other men who have sex with men (GBMSM) and transgender women (TGW) were assessed for acceptability, feasibility, and linkage to antiretroviral therapy (ART) and preexposure prophylaxis (PrEP) services.

**Methods:**

Between April and August 2019, peer mobilizers mobilized clients by offering HIV oral self-tests and immediate clinic referral for clients with AEHI symptoms. Mobilized participants received clinic-based rapid antibody testing and point-of-care HIV RNA testing. Newly diagnosed participants including those derived from HIV testing services were offered immediate ART and HPN. Partners were recruited through HPN.

**Results:**

Of 772 mobilized clients, 452 (58.5%) enrolled in the study as mobilized participants. Of these, 16 (3.5%) were HIV newly diagnosed, including 2 (0.4%) with AEHI. All but 2 (14/16 [87.5%]) initiated ART. Thirty-five GBMSM and TGW were offered HPN and 27 (77.1%) accepted it. Provider referral identified a higher proportion of partners tested (39/64 [60.9%] vs 5/14 [35.7%]) and partners with HIV (27/39 [69.2%] vs 2/5 [40.0%]) than index referral. Of 44 enrolled partners, 10 (22.7%) were newly diagnosed, including 3 (6.8%) with AEHI. All 10 (100%) initiated ART. PrEP was initiated among 24.0% (103/429) mobilized participants and 28.6% (4/14) partners without HIV.

**Conclusions:**

HPN, combined with a peer mobilization–led self-testing strategy and AEHI screening for GBMSM and TGW, appears to be acceptable and feasible. These strategies, especially HPN provider referral, effectively identified undiagnosed HIV infections and linked individuals to ART and PrEP services.

## Teaser

HIV partner notification services, combined with a peer mobilization–led self-testing strategy and acute and early HIV infection screening for gay, bisexual, and other men who have sex with men and transgender women effectively identified undiagnosed HIV infections in coastal Kenya.

Gay, bisexual, and other men who have sex with men (GBMSM) and transgender women (TGW) have high human immunodeficiency virus (HIV) incidences in sub-Saharan Africa (SSA) [1–3]. Recent work in the Kenyan coast demonstrated an incidence of 5.1 (95% confidence interval [CI], 2.6–9.8) per 100 person-years (PY) among GBMSM and 20.6 (95% CI, 6.6–63.8) per 100 PY among TGW [1]. However, GBMSM and TGW are often not engaged in HIV prevention and care services [4, 5].

HIV partner notification services (HPN) have great potential in identifying undiagnosed HIV infections [6–9]. A recent Kenyan trial showed that HPN were safe and increased HIV testing and case-finding among heterosexual people [10]. The benefits of HPN for GBMSM in well-resourced settings have been well described [6, 11], but to the best of our knowledge, no data exist on HPN for GBMSM and TGW in SSA.

In a recent pilot study in coastal Kenya, 8.7% of GBMSM and TGW who received an HIV oral self-test from peer mobilizers were newly diagnosed with HIV [12]. Community-led HIV testing and counseling services (HTC), including provision of self-tests, combined with HPN, proved feasible and effective in diagnosing HIV and linking clients to HPN and care among key populations in Thailand [13]. However, HIV oral self-tests and other antibody-based HIV self-tests will miss acute or early HIV infection (AEHI) [14], while undiagnosed AEHI could be an important driver of the ongoing HIV epidemic among GBMSM and TGW in SSA [15–17].

We recently conducted a systematic review and meta-analysis of studies reporting strategies to mobilize GBMSM for AEHI testing [18]. We found that targeted AEHI testing (ie, testing of individuals with certain behavioral factors or symptoms) resulted in substantially higher AEHI yields than universal AEHI testing, and that AEHI yield may be increased by using published behavioral and/or symptom scores [19–21]. Considering the costs of HIV RNA testing, required for AEHI diagnosis [15, 22], increasing AEHI yield through targeted testing may be an efficient strategy in less-resourced settings. However, no AEHI yield has been reported from screening GBMSM or TGW with published AEHI behavioral and/or symptom scores globally [18].

We hypothesized that provision of HPN, combined with a peer mobilization–led self-testing strategy and AEHI screening engaging GBMSM and TGW, would increase HIV case-finding and provision of preexposure prophylaxis (PrEP) and could therefore reduce HIV transmission. However, data on the acceptability, feasibility, safety, and effectiveness of these approaches among GBMSM and TGW in SSA are lacking. The primary objective of this study was to assess whether HPN offered to GBMSM and TGW was acceptable, feasible, and safe. The secondary objectives were to assess which referral strategy (provider vs index) had the highest yield of partner testing and identifying newly diagnosed partners; the yield of HIV RNA testing of self-tested negative participants in this high-incidence population; if the AEHI testing yield could be increased through behavioral and/or symptom score screening; and the care and prevention cascade indicators among participants identified through HPN, peer mobilization, and AEHI screening.

## METHODS

### Study Setting

The study was conducted from April through August 2019 at 2 sites in coastal Kenya: Malindi Sub-County Hospital, to which the Kenya Medical Research Institute–Wellcome Trust Research Programme (KEMRI-KWTRP) has been providing support in HTC to GBMSM and TGW [1]; and the KEMRI-KWTRP research clinic Mtwapa, with longstanding experience on HIV studies among key populations [23, 24]. Both sites (approximately 100 km apart) work in close collaboration with local community–based lesbian, gay, bisexual, transgender, queer, intersex organizations.

### Patient Consent Statement

All participants provided written informed consent prior to enrollment and received the equivalent of US$3.50 for travel reimbursement. The KEMRI Scientific Ethical Review Unit approved the study (135/3747).

### Study Design and Population

The 2 recruitment strategies were peer mobilization and HPN. Participants were 18 years or older, male sex assigned at birth, and reporting oral or anal sex with a man in the previous 6 months or a sexual partner of a participant with HIV. Detailed eligibility criteria are reported in Supplementary Table 1.

### Peer Mobilization

For the peer mobilization recruitment strategy, GBMSM (n = 14) and TGW (n = 13) peer mobilizers mobilized clients behaviorally vulnerable to HIV or with AEHI or sexually transmitted infection (STI) symptoms, based on 2 published screening scores (Supplementary Table 1) [23, 25], aiming to recruit GBMSM and TGW with undiagnosed HIV. They distributed OraQuick HIV oral self-tests within their networks and referred clients for study screening, regardless of the self-test result. Since the antibody-based self-test performs poorly in diagnosing AEHI [14], peer mobilizers gave clients with AEHI symptoms a symptom referral card and immediately referred them for enrollment and further testing. Peer mobilizers were compensated with US$15 in each week that they achieved their target of mobilizing 5 clients for study screening. Additionally, peer mobilizers received US$5 weekly for travel reimbursement. No target was set for enrollment after immediate referral based on symptoms, as we were uncertain how many symptomatic clients the peer mobilizers would meet during their mobilizations.

#### Study Procedures—Mobilized Participants

When GBMSM and TGW presented to the study clinic after mobilization in the community, eligibility for enrollment was assessed and a behavior and symptom screening score completed (Supplementary Table 1) [23, 25]. After study enrollment and regardless of the self-test result, participants were tested by a health care provider (HCP) using a rapid antibody test (Alere Determine, Abbott). If this test was negative, a point-of-care qualitative HIV RNA test (GeneXpert, Cepheid) was performed. If either the rapid antibody test or the qualitative HIV RNA test was positive, a second rapid antibody test (First Response, Premier Medical Corporation) was performed. AEHI was defined as a positive qualitative HIV RNA result and a negative or discrepant (ie, 1 test positive and 1 negative) rapid antibody result; or as 2 positive rapid antibody results and a self-reported negative HIV test in the previous 3 months. Newly diagnosed participants were offered immediate antiretroviral therapy (ART) free of charge and HPN, and participants without HIV were offered PrEP free of charge.

### HIV Partner Notification Services

All newly diagnosed GBMSM and TGW mobilized in the community, their partners diagnosed through HPN, and clients diagnosed through routine HTC at 1 of the study clinics were offered HPN. Those accepting HPN were enrolled as index participants.

#### Study Procedure—Index Participants

An HCP interviewed index participants about their sexual partners in the previous 12 months, according to Kenyan guidelines [26]. If the index participant reported any risk of intimate partner violence within a partnership, this partner was not notified. HPN safety was assessed among index participants willing to return to the study clinic 1 month after initiating HPN.

#### Study Procedures—HIV Partner Notification Services

For each partner, the HCP and index participant agreed upon the best HPN strategy: provider referral or index referral. In both strategies, a self-test could be provided to the partner. In most cases, HCP contacted partners by phone. Initially, the World Health Organization–recommended message “You might have been exposed to HIV” was used [27]. However, this did not solicit partners coming forward for testing. The message “We would like to discuss important health-related issues” was received better. Approximately 2–3 phone calls were made to build rapport before partners presented for enrollment. When phone contact could not be established, peer mobilizers supported HCP and index participants by using their knowledge of sexual networks and meeting places (ie bars, restaurants, or common outdoor locations). For index referred partners who did not present at the study clinic, HCP and peer mobilizers followed up with the index participant and, if needed, supported the index participant in HPN. Partners notified by an HCP were classified as provider referral, regardless of the initial strategy.

#### Study Procedures—Partners

After partner enrollment, the study procedures for partners followed those of mobilized participants. Among partners who reported to be known positive, engagement in HIV care was assessed and, if needed, partners were counseled on re-engagement.

### HIV Care and Prevention Cascades

To assess the HIV care cascade, we retrospectively conducted quantitative HIV RNA testing (GeneXpert) on all samples that tested positive with qualitative HIV RNA or rapid antibody tests. We defined viral suppression as viral load <50 copies/mL. Newly diagnosed established infection was defined as 2 positive rapid antibody results, a viral load ≥50 copies/mL, and the participant reporting not to be previously aware of their status. Known positive was defined as 2 positive rapid antibody results and a viral load <50 copies/mL or a participant reporting to be known positive, regardless of their viral load. The HIV prevention cascade indicator included the proportion of participants without HIV initiating PrEP.

### Statistical Analysis

We conducted a descriptive analysis using Stata version 15.1 software of demographic characteristics, behavioral factors, and symptoms among mobilized participants and partners, HIV testing and yield (stratified by infection stage and by provider vs index referral strategy), self-test experience, HPN acceptability and safety, and the ratio of partners enrolled in the study per index participant. We also assessed the number of participants needed to contact (NNC) and number needed to test (NNT) to enroll 1 newly diagnosed participant and 1 participant with unsuppressed viral load, and care and prevention cascade indicators. To assess whether AEHI yield could be increased by using behavioral and/or symptom score screening, we applied 2 published scores [23, 25] and calculated AEHI yield for each score cutoff (ie, the proportion of AEHI cases among participants with a score above the cutoff) and used exact binomial 95% confidence intervals (CIs).

## RESULTS

### Peer Mobilization

In total, 772 clients were mobilized by peer mobilizers. Clients received a self-test (n = 584) or an immediate symptom referral card (n = 188) (Supplementary Figure 1). Of these, 68.5% (529/772; 521 self-test referrals and 8 symptom referrals) presented at a study clinic, and 85.4% (452/529; 444 self-test referrals and 8 symptom referrals) were eligible for study enrollment and received clinic-based HTC.

#### Characteristics of Mobilized Participants

Median age was 26 (interquartile range [IQR], 22–30) years; Supplementary Table 2). Three hundred forty-seven of 451 (76.9%) participants reported meeting their sexual partners in a bar or restaurant, 250 of 451 (55.4%) met outdoors, and 64 of 451 (14.2%) used social media to meet partners. Facebook was the most frequently reported social media platform (57/64 [89.1%]). Of 445 participants, 23 (5.2%) had never tested for HIV, while 31 (7.0%) reported a last HIV test in the previous 3 months. Participants with HIV more frequently reported receptive anal sex than participants without HIV (34.8% vs 11.7%), any AEHI symptom (17.4% vs 8.4%), or any STI symptom (17.4% vs 1.4%).

#### HIV Testing, Care, and Prevention Cascade

Of the 452 mobilized participants who were enrolled, 16 (3.5%) were newly diagnosed, including 2 (0.4%) with AEHI. The NNC and NNT to enroll 1 newly diagnosed participant were 48.3 (772/16) and 28.3 (452/16), respectively. Fourteen (87.5% [2/2, 100% with AEHI]) initiated ART following a median of 2 (IQR, 0–15) days; 1.6% (7/452) were known positive. Of the 429 mobilized participants without HIV, 103 (24.0%) initiated PrEP following a median of 1 (IQR, 0–4) day. Most frequently reported reasons for not initiating PrEP were dislike of medication (151/325 [46.5%]), fear of side effects (49/325 [15.1%]), needing time to decide (29/325 [8.9%]), not knowing much about PrEP (23/325 [7.1%]), and fear of stigma (17/325 [5.2%]).

#### Self-Test Experience

The majority of mobilized participants did their self-test at the study clinic (343/443 [77.4%]) (Supplementary Table 3). When asked on a 4-point Likert scale, 419 of 443 (94.6%) were “very satisfied” with the self-test process and 442 of 444 (99.8%) would recommend self-testing to a friend or family member.

### HIV Partner Notification Services

#### Index Participants

**Figure 1.**
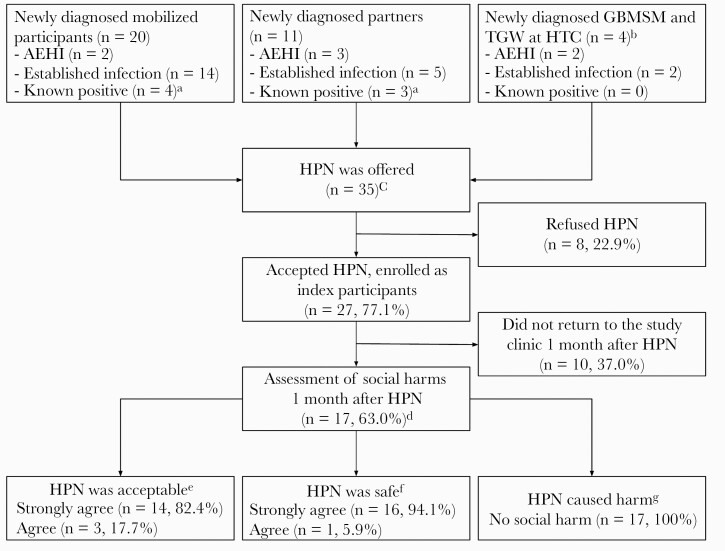
Enrollment of index participants in human immunodeficiency virus (HIV) partner notification services (HPN) and the assessment of social harms among gay, bisexual, and other men who have sex with men (GBMSM) and transgender women (TGW) in coastal Kenya, April–August 2019. ^a^For 4 newly diagnosed mobilized participants and 3 newly diagnosed partners who reported to be HIV negative at study enrollment, the rapid antibody results were positive. As these participants were classified as newly diagnosed, they were offered HPN. However, their retrospective quantitative HIV RNA result was <50 copies/mL; they were therefore classified as known positive and presumably on suppressive antiretroviral therapy, after completion of data collection. ^b^Four participants were included in the study as they were newly diagnosed during the study period and enrolled as index participants in order to notify their partners. ^c^HPN was offered to all GBMSM and TGW who were considered newly diagnosed during the study period, regardless of the recruitment strategy. ^d^Index participants who were willing returned to the study 1 month after HPN to assess social harms. ^e^Assessed in a face-to-face standardized interview as follows: “I consider HPN an acceptable method to notify sexual partners for HIV: strongly agree, agree, disagree, strongly disagree.” ^f^“I consider HPN as a safe method to notify sexual partners for HIV: strongly agree, agree, disagree, strongly disagree.” ^g^“Did you experience any harms resulting HPN? No; relationship dissolution; loss of economic support; loss of custody of children; loss of client; change of residence; disclosure of HIV status to others; disclosure of sexuality to others; other, specify.” Abbreviations: AEHI, acute or early human immunodeficiency virus infection; GBMSM, gay, bisexual, and other men who have sex with men; HPN, human immunodeficiency virus partner notification services; HTC, human immunodeficiency virus testing and counseling services; TGW, transgender women.

Sixteen newly diagnosed mobilized participants, 8 newly diagnosed partners, and 4 clients newly diagnosed at the HTC were offered HPN (Figure 1). In addition, 4 mobilized participants and 3 partners who were initially classified as newly diagnosed and offered HPN were reclassified as known positive based on retrospective quantitative HIV RNA results being <50 copies/mL. Of these 35 participants offered HPN, 27 (77.1%) accepted HPN and were enrolled as index participants. Safety was assessed 1 month after initiating HPN among 17 of 27 (63.0%; 2 AEHI, 13 newly diagnosed, and 2 known positive) index participants: all 17 agreed or strongly agreed that HPN was an acceptable and safe method to notify partners. They did not report any harm resulting from HPN (17/17 [100%]).

#### HIV Partner Notification Services

**Figure 2.**
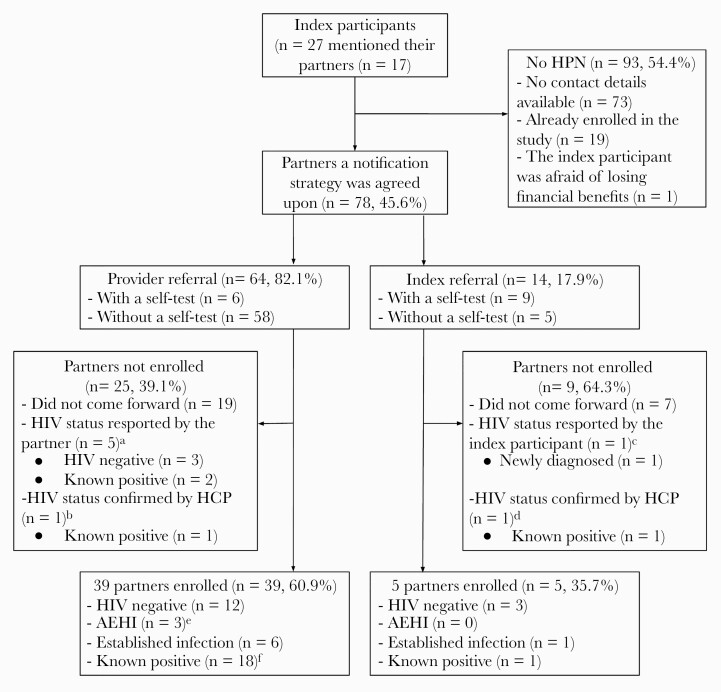
Human immunodeficiency virus (HIV) partner notification services outcomes among index participants and their partners in coastal Kenya, April–August 2019. ^a^Three partners preferred to test elsewhere and reported to have tested HIV negative; 2 partners reported to be known positive and were not interested in study enrollment. ^b^One known positive partner was already in HIV care at the study clinic and was not interested in study enrollment. ^c^The index participant reported that his partner tested positive after using a self-test; however, the partner was not interested in confirmatory testing, study enrollment, or linkage to HIV care. ^d^The partner presented for HIV testing to the study clinic and was newly diagnosed, but was not interested in study enrollment. ^e^Defined as 2 positive rapid antibody tests and a self-reported negative HIV test in the previous 3 months. ^f^For 3 partners with positive rapid antibody results who reported to be HIV negative at study enrollment, the retrospective quantitative HIV RNA result was <50 copies/mL; they were therefore classified as known positive. Abbreviations: AEHI, acute or early human immunodeficiency virus infection; HCP, health care provider; HIV, human immunodeficiency virus; HPN, human immunodeficiency virus partner notification services.

The 27 index participants reported having had 171 sexual partners in the previous 12 months (Figure 2). Of these, 8.2% (14/171) were not reported immediately after diagnosis, but rather during follow-up conversations between the HCP and index participant in the month following HPN initiation. Ninety-three of 171 (54.4%) partners were not notified, mainly (73/171 [42.7%]) because the index participant did not have any contact details of the partner. Furthermore, 19 of 171 (20.4%) were mentioned by multiple index participants and were already enrolled in the study. For the 78 partners for which a notification strategy was agreed upon, the index participant reported no risk of intimate partner violence. Among the 78 partners, 64 (82.1%) were notified through provider referral and 14 (17.9%) through index referral.

#### Partners

**Figure 3.**
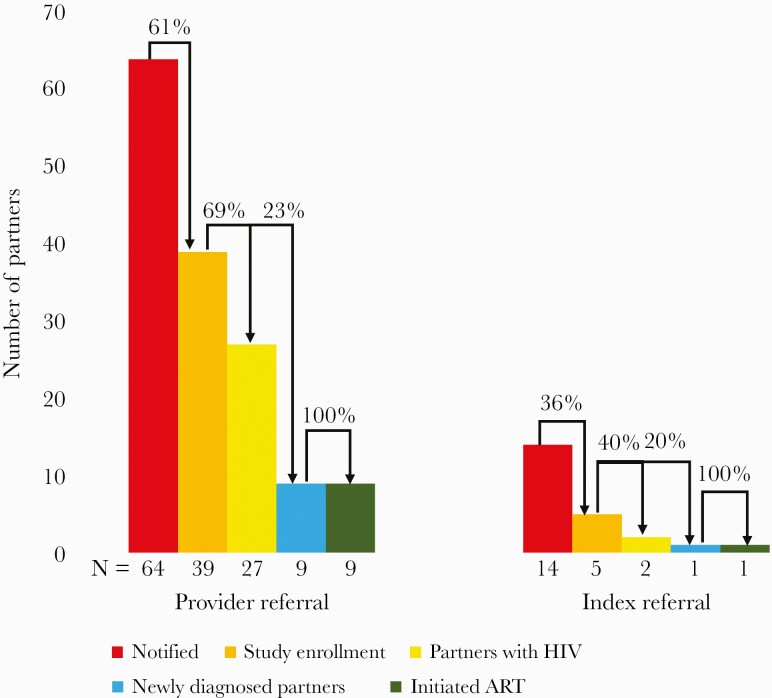
Human immunodeficiency virus partner notification outcomes in coastal Kenya, April–August 2019. Both providers and index participants were supported by peer mobilizers in notifying sexual partners. If interested, a self-test was provided to the partner. Abbreviations: ART, antiretroviral therapy; HIV, human immunodeficiency virus.

In total, 56.4% (44/78) of notified partners were enrolled. The ratio of partners enrolled per index participant was 1.63 (44/27). Characteristics of partners are displayed in Supplementary Table 4. Provider vs index referral identified a higher proportion of partners tested (39/64 [60.9%] vs 5/14 [35.7%]), partners with HIV (27/39 [69.2%] vs 2/5 [40.0%]), and partners newly diagnosed with HIV (9/39 [23.1%] vs 1/5 [20.0%]) (Figure 3). Of 44 enrolled partners, 10 (22.7%) were newly diagnosed, including 3 (6.8%) with AEHI. Median viral load was 4.9 (IQR, 4.4–5.2) log_10_ copies/mL among partners with established infection and 5.6 (IQR, 4.6–5.7) log_10_ copies/mL among partners with AEHI. The NNC and NNT to enroll 1 newly diagnosed partner were 7.8 (78/10) and 4.4 (44/10), respectively; and the NNC and NNT to enroll 1 partner with unsuppressed viral load were 6.5 (78/12) and 3.7 (44/12), respectively. All 10 (100%) newly diagnosed partners initiated ART after a median of 1 (IQR, 0–2) day. Among the partners without HIV, 28.6% (4/14) initiated PrEP.

### Acute and Early HIV Infection Yield

We assessed AEHI yield for 2 published screening scores: 1 behavioral score including 5 factors [23] and 1 symptom score including 7 factors [25]. When combining mobilized participants and partners, all 5 AEHI cases were identified by the behavioral score and 3 by the symptom score (Table 1). AEHI yield increased with an increasing cutoff of the behavioral score: from 1.1% (95% CI, .03–2.4) at a cutoff of ≥1 to 3.7% (95% CI, .09–19.0) at a cutoff of ≥4, while the proportion of participants requiring AEHI testing decreased from 100% to 5.7%, respectively. Similarly, AEHI yield increased with an increasing cutoff of the symptom score: from 0.8% (95% CI, .2–2.5; cutoff of ≥1) to 20.0% (95% CI, .5–71.6; cutoff of ≥4), while the proportion of participants requiring AEHI testing decreased from 74.2% to 1.1%. However, both scores only identified 20.0% (1/5) of AEHI cases at a cutoff of ≥4.

**Table 1.** Screening and Yield of Acute and Early HIV Infections Among Mobilized Participants and Their Partners in Coastal Kenya, April–August 2019

Participants and Score	AEHI Cases^a^	Participants With a Score of at Least the Cutoff	AEHI Yield	(95% CI)	% Requiring AEHI Testing
Mobilized participants^b^					
No.	2	448			
Behavioral score^c^ ≥1	2	448	0.4%	(.05–1.5)	100%
Behavioral score ≥2	1	292	0.3%	(.009–1.9)	65.2%
Behavioral score ≥3	1	87	1.1%	(.03–6.2)	19.4%
Behavioral score ≥4	0	22	0%	(0–.15)^d^	4.9%
Behavioral score ≥5	0	0	0%	NA	0%
Symptom score^e^ ≥1	1	335	0.3%	(.008–1.7)	74.8%
Symptom score ≥2	1	37	2.7%	(.07–14.2)	8.3%
Symptom score ≥3	1	18	5.6%	(.1–27.3)	4.0%
Symptom score ≥4	1	5	20.0%	(.5–71.6)	1.1%
Symptom score ≥5	1	1	100%	(2.5-1)^d^	0.2%
Symptom score ≥6	0	0	NA	NA	0%
Mobilized participants and partners combined^f^					
No.	5	476			
Behavioral score^c^ ≥1	5	476	1.1%	(.03–2.4)	100%
Behavioral score ≥2	3	312	1.0%	(.2–2.8)	65.5%
Behavioral score ≥3	3	102	2.9%	(.6–8.4)	21.4%
Behavioral score ≥4	1	27	3.7%	(.09–19.0)	5.7%
Behavioral score ≥5	0	1	0%	(0–.975)^d^	0.2%
Symptom score^e^ ≥1	3	353	0.8%	(.2–2.5)	74.2%
Symptom score ≥2	1	38	2.6%	(.07–13.8)	8.0%
Symptom score ≥3	1	18	5.6%	(.01–27.3)	3.8%
Symptom score ≥4	1	5	20.0%	(.5–71.6)	1.1%
Symptom score ≥5	1	1	100%	(.025–1)^d^	0.2%
Symptom score ≥6	0	0	NA	NA	0%

Abbreviations: AEHI, acute or early human immunodeficiency virus infection; CI, confidence interval; NA, not accessible.

^a^Defined as a positive qualitative HIV RNA test and discrepant rapid antibody tests (n = 1) or 2 positive rapid antibody tests and a self-reported negative HIV test in the previous 3 months (n = 4).

^b^Four missing values.

^c^Score range 0–5: age 18–24 years (score of 1); in the previous 7 days: any condomless sex (score of 1); in the previous 3 months: sex with men only (score of 1), receptive anal sex (score of 1), group sex (score of 1).

^d^One-sided 97.5% CI.

^e^Score range 0–9: age 18–29 years (score of 1); in the previous 14 days: self-reported fever (score of 1), diarrhea (score of 1), fatigue (score of 1), body ache (score of 1), sore throat (score of 1), or genital ulcer (3 points).

^f^Twenty missing values, as behavioral factors and symptoms were not assessed for partners who reported to be known positive.

### HIV Care Cascade

**Figure 4.**
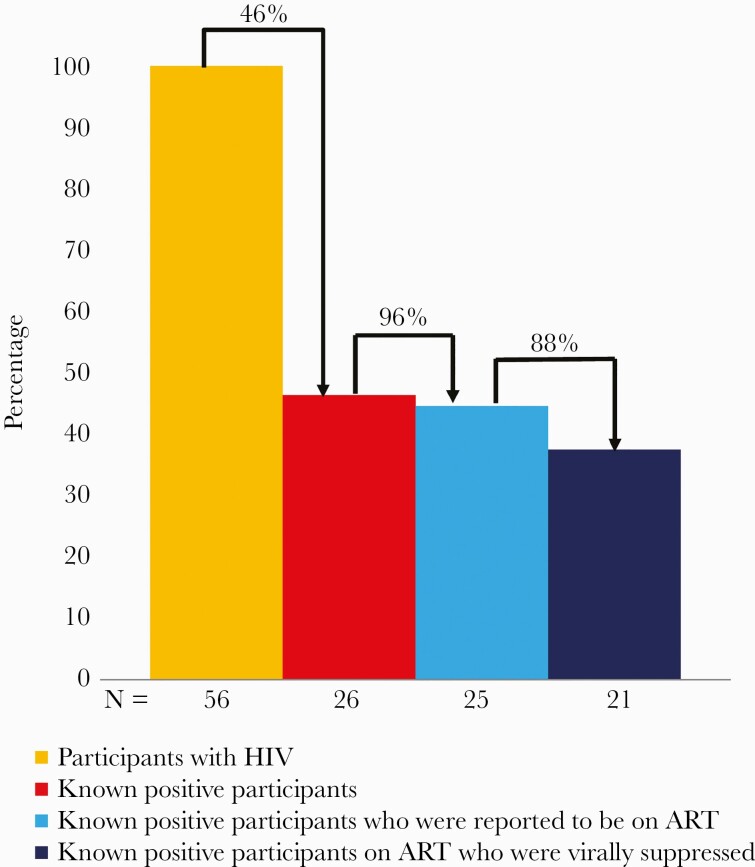
The human immunodeficiency virus (HIV) testing and care cascade among mobilized participants and their partners in coastal Kenya, April–August 2019. The 56 participants with HIV were recruited through oral self-test referrals (n = 22), immediate symptom referral cards (n = 1), HIV partner notification services (n = 29), and routine HIV testing and counseling at the study clinics (n = 4). The latter 4 were included in the study as they were newly diagnosed gay, bisexual, and other men who have sex with men or transgender women during the study period and enrolled as index participants in order to notify their partners. Abbreviations: ART, antiretroviral therapy; HIV, human immunodeficiency virus.

Among the 56 participants with HIV, 30 (53.6%) were newly diagnosed and 26 (46.4%) were known to be positive (Figure 4). Of these 26, 25 (96.2%) were reported to be on ART and 21 of 24 (87.5%) were virally suppressed. Median viral load among the 3 known positive participants not virally suppressed was 4.7 log_10_ copies/mL.

## DISCUSSION

In this study, HPN offered to GBMSM and TGW in coastal Kenya appeared acceptable, feasible, and safe. Using a peer mobilization–led self-testing strategy, 48 clients needed to be contacted and 28 clients needed to be tested to identify 1 newly diagnosed participant. With HPN, 8 partners needed to be contacted and 4 partners needed to be tested to identify 1 newly diagnosed partner. We found a high ratio (1.63) of partners enrolled per index participant. Recent studies offering HPN to GBMSM reported ratios ranging from 0.56 in Thailand [13], 0.64 in Guangzhou [28], and 0.71 in the Netherlands [29] to 1.11 in San Diego [6]. The high ratio of partners tested in this study may be explained by the support HCP received from peer mobilizers in notifying partners and the experience of the study teams working with key populations in the same areas over the past 10 years. Another explanation could be that we recruited participants from an interconnected community who knew each other fairly well, as 1 in 5 partners mentioned were already enrolled in the study. Most partners were notified through intense HCP follow-up, supported by peer mobilizers’ understanding of sexual networks and meeting places of GBMSM and TGW. This support may have amplified the effectiveness of HPN.

HCP built rapport with both index participants and partners and delivered culturally sensitive messages, which were adjusted during the study. Index participants elicited an additional 8.2% of their partners during follow-up conversations with an HCP in the month following HPN, suggesting the need for building trust between HCP and index participants before full disclosure of sexual partners.

Provider referral resulted in a higher proportion of partners tested and being newly diagnosed than index referral, in line with previous studies [10, 29–32]. This underlines the importance of the role of HCP in HPN, even in a context where GBMSM and TGW are criminalized and highly stigmatized and may be reluctant to share details about sexual partners [33, 34]. Importantly, provider referral identified 3 partners with AEHI, all having a high viral load, confirming the high AEHI yield following HPN described in San Diego [6].

Our study confirms the value of HIV RNA testing in a high HIV incidence population [35, 36]. We confirmed AEHI in 1 (1/452 [0.2%]) mobilized participant with discrepant rapid antibody results, which facilitated immediate ART initiation. Kenyan guidelines recommend retesting 2 weeks after initial discrepant rapid antibody results, thus potentially delaying ART initiation in people with AEHI [26]. Our data suggest that if a more stringent cutoff of the behavioral or symptom score would be used to select people for HIV RNA testing, the AEHI yield could be up to 3.7% (behavioral score) or 20.0% (symptom score), while only offering HIV RNA testing to a small subset of participants. This is consistent with findings from our recently conducted meta-analysis, in which the pooled estimate for AEHI screening targeted to behaviorally vulnerable or symptomatic GBMSM was 11.1% (95% CI, 5.9%–17.6%) [18]. However, using a higher cutoff will inevitably miss a substantial proportion of AEHI cases and should only be used when resources are constrained. Furthermore, AEHI yields may not be generalizable as study participants likely have derived from a closed network.

Data on the HIV care cascade indicators among GBMSM and TGW in SSA are scarce, but estimated to be well below the Joint United Nations Programme on HIV/AIDS targets [37]. Our data suggest that among known positive participants, care cascade indicators met the second and third “90-90-90” indicators [38]. In contrast, only 46.4% of participants were aware of their HIV infection. Furthermore, only 7.0% of mobilized participants and 29.2% of partners reported having had an HIV test in the previous 3 months. Kenyan guidelines recommend HIV testing every 3 months for GBMSM and TGW [26]. Our study, which captured a high number of AEHI also among partners, confirms that GBMSM and TGW should test quarterly. Frequent testing should also be delivered through self-testing, as our participants reported remarkably high satisfaction (>99%) with the self-test process. This is further supported by previous studies, showing that self-testing promotes HIV testing uptake, particularly if distributed by the community [13, 39–41]. Mobilized participants with HIV reported STI symptoms more frequently than participants without HIV (17.4% vs 1.4), demonstrating that a mobilization strategy focusing on clients reporting STI symptoms will likely increase the HIV testing yield.

Limitations include no follow-up outcomes of participants who initiated PrEP and ART; therefore, we were unable to assess retention in care. Second, there was a substantial loss of (potential) participants in multiple steps of the study, making it uncertain if the success of the study was due to client self-selection, the clinics’ longstanding relationship with the community, or the strategies studied. For example, even though 188 symptom referral cards were distributed, only 8 participants enrolled in the study following immediate symptom referral. This may have resulted from the lack of a weekly target for immediate symptom referrals for peer mobilizers and/or the complexity of distributing self-tests and symptom referral cards at the same time. Furthermore, while all 27 index participants were invited to return to the study clinic to assess HPN safety, only 17 did. Third, newly diagnosed female partners (n = 2) were not eligible for enrollment as index participants, as the study focused on GBMSM and TGW sexual networks. This may have led to missed diagnoses among their partners. Fourth, results may be difficult to generalize, given the close GBMSM and TGW networks in these relatively small coastal towns. Last, the intense provider follow-up of index participants and partners, as well as supervision of peer mobilizers, was highly time consuming and may be difficult to scale up.

## CONCLUSIONS

This study is the first to report on HPN offered to GBMSM and TGW in SSA. In addition, this is the first study reporting an AEHI yield following targeted AEHI screening using published screening scores. Our findings suggest that HPN, combined with a peer mobilization–led self-testing strategy and AEHI screening for GBMSM and TGW, appears to be acceptable, feasible, and safe. These strategies, in particular HPN provider referral, effectively identified undiagnosed HIV infections, including a high proportion of AEHI among partners, and linked individuals to ART and PrEP services. The majority of partners were notified through intense provider follow-up, supported by peer mobilizers, which enabled HIV testing. Delivering culturally tailored messages and building rapport with partners was an ongoing process necessary to achieve partners coming forward for HIV testing. The very high satisfaction of oral self-testing suggests the utility of this strategy in future programs. Future studies should focus on the generalizability and scalability of this approach, the cost-effectiveness of targeted AEHI screening with HIV RNA testing, and the effectiveness of strategies increasing regular HIV testing among GBMSM and TGW in SSA.

## Supplementary Data

Supplementary materials are available at *Open Forum Infectious Diseases* online. Consisting of data provided by the authors to benefit the reader, the posted materials are not copyedited and are the sole responsibility of the authors, so questions or comments should be addressed to the corresponding author.

ofab219_suppl_Supplementary_Materials

## Notes

***Author contributions.*** M. D., S. M. G., E. M. E., and E. J. S. designed the study. M. D., K. M., A. K., T. M., S. M., A. W., N. M., T. O., E. G., S. P., and E. M. E. conducted the study and collected the data. M. D. and T. O. cleaned the data. M. D. conducted the statistical analysis and drafted the manuscript. M. F. S. L. and E. J. S. had oversight in the statistical analysis. E. J. S. provided overall oversight for fieldwork, supervision, and manuscript writing. All authors critically reviewed and revised the manuscript and approved the final version for publication.

***Acknowledgments.*** We would like to acknowledge all participants who took part in this study. We further acknowledge the peer mobilizers in Malindi and Mtwapa who were invaluable for mobilizing participants and their support in HIV partner notification services, and the following lesbian, gay, bisexual, transgender and intersex organizations involved in the study: Malindi Desire Initiative, AMKENI Malindi, HAPA Kenya, PEMA Kenya, GALCK, and ISHTAR MSM Health and Social Wellbeing. We would also like to acknowledge the Kenya Medical Research Institute (KEMRI)–Wellcome Trust Research Programme clinic staff who made this study possible: Peter Mugo, Bernadette Kombo, George Makau, Makobu Kimani, Clara Agutu, Dan Otieno, Alexander Thiong’o, Oscar Chirro, Jennifer Kanungi, Margaret Juma, Amin Hassan, Joakim Esbjornsson, Joseph Nzioka, Angela Omore, Kelly Ramko, John Mwambi, Lucie Ikumi, Esther Ndiang’ui, Riziki Rodgers, Raymond Nzaro, John Omoi, Eshter Yaa, Clara Karani, Brenda Nekesa, Francisca Mwangiri, Leonita Myongesa, Maxwell Ong’aro, Beatrice Wavinya, Fred Ogada, Elizabeth Wahome, Cynthia Abdi, Fanice Kiteki, Rufus Gathitu, Lucy Charo, Winston Kazungu, and Pamela Anyango.

***Disclaimer.*** The contents are the responsibility of the study authors and do not necessarily reflect the views of the US Agency for International Development (USAID), the National Institutes of Health (NIH), the US or UK governments, the African Academy of Sciences (AAS), the New Partnership for Africa’s Development Planning and Coordinating Agency (NEPAD), or the Wellcome Trust. This report was published with permission from the director of KEMRI.

***Financial support.*** This work was supported by the International AIDS Vaccine Initiative (IAVI) and the KEMRI Wellcome Trust Research Programme at the Centre for Geographical Medicine Research–Kilifi, supported by core funding from the Wellcome Trust (number 203077). This study was made possible by the generous support of the American people through USAID. This work was also supported in part through the Sub-Saharan African Network for TB/HIV Research Excellence, a DELTAS Africa Initiative (DEL-15-006). The DELTAS Africa Initiative is an independent funding scheme of the AAS Alliance for Accelerating Excellence in Science in Africa and is supported by NEPAD with funding from the Wellcome Trust (grant number 107752) and the UK government. E. J. S. receives research funding from IAVI, NIH (grant number R01AI124968) and the Wellcome Trust. M. D. receives funding through a PhD Scholarship from the Graduate School of Amsterdam UMC–Academic Medical Center.

***Potential conflicts of interest.*** All authors: No reported conflicts of interest.
