# Supplementary material for: Peer Mobilization and Human Immunodeficiency Virus (HIV) Partner Notification Services Among Gay, Bisexual, and Other Men Who Have Sex With Men and Transgender Women in Coastal Kenya Identified a High Number of Undiagnosed HIV Infections
Source: Open Forum Infect Dis. 2021 Apr 29;8(6):ofab219. doi: 10.1093/ofid/ofab219 (PMC8186249; doi:10.1093/ofid/ofab219)
Supplement: ofab219_suppl_Supplementary_Materials [file ofab219_suppl_supplementary_materials.pdf]

Supplementary Table 1. Eligibility criteria of mobilised participants, index participants and their partners

**Study population**	**Eligibility criteria**
Mobilised participants	Inclusion criteria:<br>- Age 18 years or older;<br>- Male sex assigned at birth;<br>- Reporting oral or anal sex with men in the previous six months;<br>- HIV negative or of unknown HIV status before doing the self-test;<br>- Willing to provide a blood sample;<br>- Reporting at least one behavioural factor or at least two AEHI symptoms or at least one STI symptom[a]<br>• Behavioural factors: in the previous seven days: any condomless sex; or in the previous three months: sex with only men, receptive anal sex or group sex;<br>• AEHI symptoms: in the previous 14 days: fever, diarrhoea, fatigue, body aches, sore throat;<br>• STI symptoms: in the previous 14 days: genital ulcer, genital discharge or anal discharge.<br>Exclusion criteria:<br>- Well-adherent to PrEP
Index participants	Inclusion criteria:<br>- Age 18 years or older;<br>- Male sex assigned at birth;<br>- Reporting oral or anal sex with men in the previous six months;<br>- Being newly diagnosed with HIV, either after peer mobilisation, AEHI screening, HPN or at the HTC of one of the study clinics during the study period (April through August 2019);<br>- Willing to provide a blood sample.
Partners	Inclusion criteria:<br>- Age 18 years or older;<br>- Notified for HIV as part of the present study;<br>- Willing to provide a blood sample.

AEHI, acute or early HIV infection; PrEP, pre-exposure prophylaxis; STI, sexually transmitted infection. a. Based on published behavioural PrEP eligibility score (Wahome E et al. An Empiric Risk Score to Guide PrEP Targeting Among MSM in Coastal Kenya. AIDS Behav. 2018;22(Suppl 1):35-44.) and AEHI symptom score (Sanders EJ et al. Targeted screening of at-risk adults for acute HIV-1 infection in sub-Saharan Africa. AIDS. 2015;29 Suppl 3:S221-30.).

Supplementary Figure 1. Study mobilisation, enrolment and HIV testing among gay, bisexual and other men who have sex with men and transgender women in coastal Kenya, April through August 2019

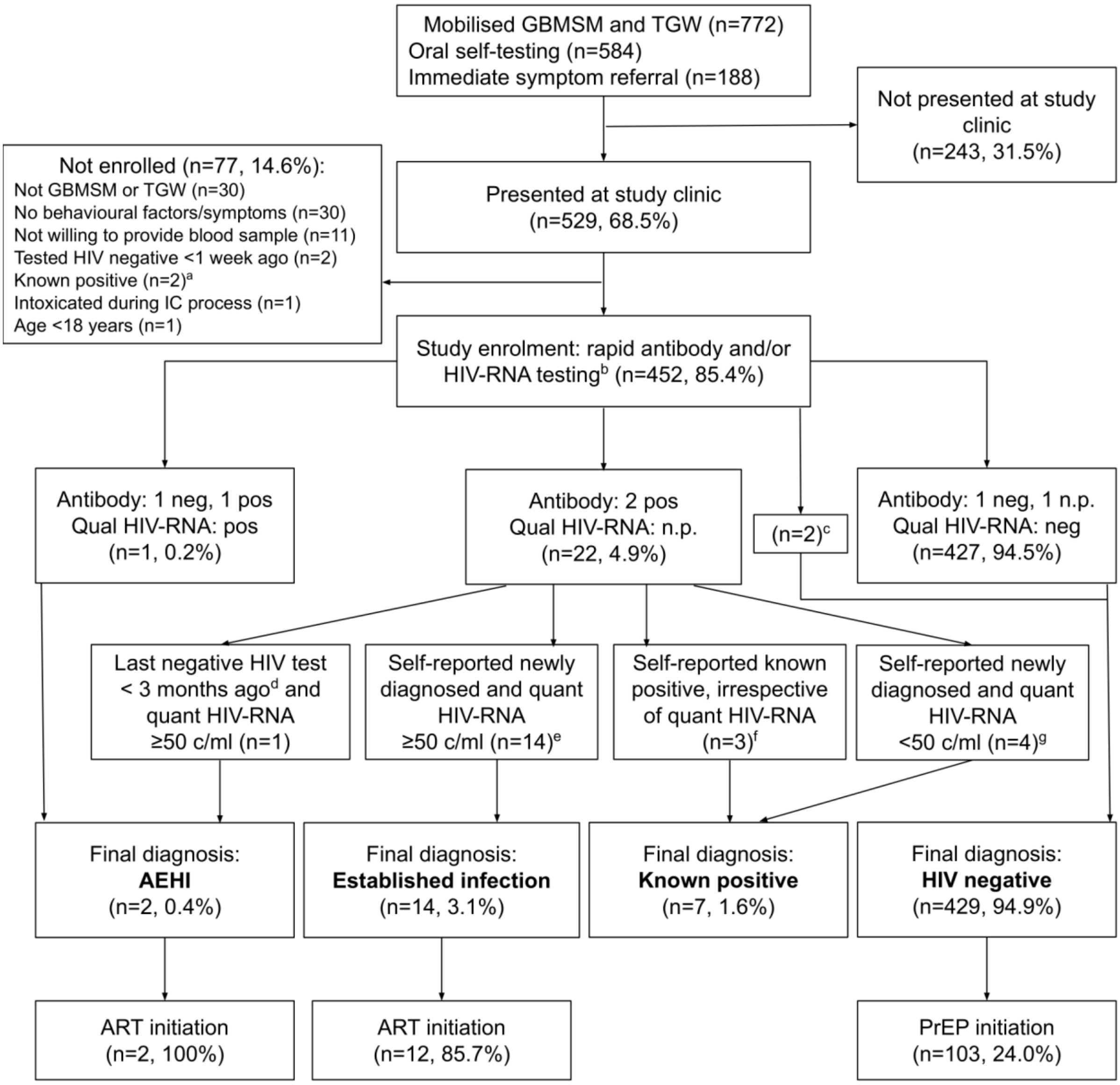

AEHI, acute or early HIV infection; ART, antiretroviral therapy; c/ml; copies per millilitre; GBMSM, gay, bisexual and other men who have sex with men; HTC, HIV testing and counselling services; IC, informed consent; neg, negative; n.p., not performed; pos, positive; PrEP, pre-exposure prophylaxis; qual, qualitative; quant, quantitative; TGW, transgender women.

a. Clients who reported to be known positive before study enrolment were excluded from study participation; b. Regardless of the oral self-test result, participants received a rapid antibody test. If this test was negative, a point-of-care quantitative HIV-RNA test was performed. If either the rapid antibody test or the HIV-RNA test were positive, a second rapid antibody test was performed. After completion of data collection, viral load was assessed retrospectively among all samples indicating HIV infection; c. One participant had one positive and one negative rapid antibody result, but the qualitative HIV-RNA result was negative. Dried-blot spot PCR was negative, therefore, the participant was classified as HIV negative; 1 participant had a positive qualitative HIV-RNA result and two negative rapid antibody results. The quantitative HIV-RNA result was negative, therefore, the participant was classified as HIV negative. They reported no prior PrEP or ART use; d. Self-reported; e. One missing quantitative HIV-RNA result, this participant reported to be newly diagnosed and was

therefore classified as having an established infection; f. Three participants reported to be HIV negative at study enrolment, but later reported to be aware of their HIV infection after the rapid antibody test results turned out positive, of these 2 had a retrospective quantitative HIV-RNA of <50 copies/ml, 1 of ≥50 copies/ml; g. For four participants who reported to be HIV negative at study enrolment, the retrospective quantitative HIV-RNA result was <50 copies/ml, they were therefore classified as known positive.

Supplementary Table 2. Characteristics of mobilised participants with and without HIV in coastal Kenya, April through August 2019

	Participants without HIV N (%)		Participants with HIV N (%)		Total N (%)	
N	429	(100%)	23	(100%)	452	(100%)
Age (years)[a,b]	25	(22-30)	29	(24-36)	26	(22-30)
Male sex assigned at birth	429	(100%)	23	(100%)	452	(100%)
Gender						
Male	421	(98.1%)	23	(100%)	444	(98.2%)
TGW / Female[c]	7	(1.6%)	0	(0%)	7	(1.5%)
Non-binary	1	(0.2%)	0	(0%)	1	(0.2%)
Sexuality[d]						
Gay / homosexual	118	(27.6%)	13	(56.5%)	131	(29.1%)
Bisexual	306	(71.7%)	10	(43.5%)	316	(70.2%)
Other[e]	3	(0.7%)	0	(0)	3	(0.7%)
Residing in the coast area since						
Less than 5 years	59	(13.8%)	1	(4.4%)	60	(13.3%)
At least 5 years	62	(14.5%)	8	(34.8%)	70	(15.5%)
Born at the coast	308	(71.8%)	14	(60.9%)	322	(71.2%)
Location / method of meeting partners[f]						
Bar or restaurant[g]	331	(77.2%)	16	(72.7%)	347	(76.9%)
Outdoors[g,h]	239	(55.7%)	11	(50.0%)	250	(55.4%)
Existing contact[g]	149	(34.7%)	11	(50.0%)	160	(35.5%)
Social media[f,g]	56	(13.1%)	8	(36.4%)	64	(14.2%)
Facebook[i]	50	(89.3%)	7	(87.5%)	57	(89.1%)
WhatsApp[i]	36	(64.3%)	7	(87.5%)	43	(67.2%)
Grindr[i]	19	(33.9%)	7	(87.5%)	26	(40.6%)
Romeo[i]	12	(21.4%)	2	(25.0%)	14	(21.9%)
Time since last HIV test[j]						
Less than 3 months	30	(7.0%)	1	(6.3%)	31	(7.0%)
From 3 months through 1 year	272	(63.4%)	10	(62.5%)	282	(63.4%)
At least 1 year	105	(24.5%)	4	(25.0%)	109	(24.5%)
Never tested for HIV	22	(5.1%)	1	(6.3%)	23	(5.2%)
Sexual behaviour						
At least one behavioural factor	429	(100%)	23	(100%)	452	(100%)
Any condomless sex[k]	371	(86.5%)	20	(87.0%)	391	(86.5%)
Sex with only men[l]	179	(41.7%)	9	(39.1%)	188	(41.6%)
Receptive anal sex[l]	50	(11.7%)	8	(34.8%)	58	(12.8%)
Group sex[l]	24	(5.6%)	1	(4.3%)	25	(5.5%)
Self-reported AEHI symptoms[m]						
At least one AEHI symptom	36	(8.4%)	4	(17.4%)	40	(8.9%)
Fever	3	(0.7%)	1	(4.3%)	4	(0.9%)
Diarrhoea	1	(0.2%)	1	(4.3%)	2	(0.4%)
Fatigue	29	(6.8%)	4	(17.4%)	33	(7.3%)
Body ache	22	(5.1%)	2	(8.7%)	24	(5.3%)
Sore throat	5	(1.2%)	1	(4.3%)	6	(1.3%)
Self-reported STI symptoms[m]						
At least one STI symptom	6	(1.4%)	4	(17.4%)	10	(2.2%)
Genital ulcer	0	(0%)	2	(8.7%)	2	(0.4%)
Genital discharge	6	(1.4%)	2	(8.7%)	8	(1.8%)
Anal discharge	1	(0.2%)	2	(8.7%)	3	(0.7%)

AEHI, acute or early HIV infection; STI, sexually transmitted infection; TGW, transgender woman.

a. Median, interquartile range; b. 4 missing values; c. Participants with male sex assigned at birth who reported a TGW or female gender identify; d. 2 missing values; e. “Basha” (“Top” in Swahili; n=1) and “Transwoman” (n=2); f. Categories were not mutually exclusive; g. 1 missing value; h. At the park, beach, street or market; i. Among participants who reported the use of social media for meeting partners; j. Excluding 7 known positive participants; k. In the previous 7 days; l. In the previous 3 months; m. In the previous 14 days.

Supplementary Table 3. Oral self-testing among mobilised participants in coastal Kenya, April through August 2019

	N	(%)
N	444	(100%)
Where did you do the self-test?[a]		
At the study clinic	343	(77.4%)
At home	88	(19.9%)
At the LGBTQI organisation	11	(2.5%)
At the beach	1	(0.2%)
Was someone else with you when you did the self-test?[a]		
Yes	327	(73.8%)
No	116	(26.2%)
With whom did you do the test?[b]		
Peer mobiliser / Person who provided the test	270	(82.6%)
Friend	43	(13.1%)
Sex partner	12	(3.7%)
Family member	2	(0.6%)
If you had the opportunity to do the self-test again, what would you prefer?[a]		
Conduct the test in private	270	(60.9%)
Conduct the test with little supervision	169	(38.1%)
Conduct the test in the presence of a counsellor	4	(0.9%)
Overall how satisfied were you with the self-test process?[a]		
Very satisfied	419	(94.6%)
Somewhat satisfied	23	(5.2%)
A little satisfied[c]	1	(0.2%)
Not satisfied at all	0	(0%)
Would you recommend self-testing to a friend or family member?[a]		
Yes	442	(99.8%)
No[c]	1	(0.2%)

LGBTQI, lesbian, gay, bisexual, transgender, queer, intersex.

a. 1 missing value; b. Among the 327 participants who had a person present while conducting the self-test; c. Of note, the participant who reported to be “A little satisfied” would recommend self-testing to a friend or family member; the participant who reported not to recommend self-testing to a friend or family member reported to be “Very satisfied” with the self-test process.

Supplementary Table 4. Characteristics of enrolled partners with and without HIV who were notified through HIV partner notification in coastal Kenya, April through August 2019

	Partners without HIV		Partners with HIV		Total	
	N	%	N	%	N	%
N	15	(100%)	29	(100%)	44	(100%)
Age (years)[a,b]	24	(22-30)	29	(25-35)	27	(24-32)
Sex at birth						
Male	15	(100%)	22	(75.9%)	37	(84.1%)
Female	0	(0%)	7	(24.1%)	7	(15.9%)
Gender identity						
Male	11	(73.3%)	19	(65.5%)	30	(68.2%)
Female	0	(0%)	7	(24.1%)	7	(15.9%)
TGW / Female[c]	4	(26.7%)	3	(10.3%)	7	(15.9%)
Sexuality						
Gay / homosexual	8	(53.3%)	14	(48.3%)	22	(50.0%)
Bisexual	5	(33.3%)	8	(27.6%)	13	(29.5%)
Heterosexual	1	(6.7%)	5	(17.2%)	6	(13.6%)
Other[d]	1	(6.7%)	2	(6.9%)	3	(6.8%)
Born at the coast area	11	(73.3%)	25	(86.2%)	36	(81.8%)
Time since last HIV test[e,f]						
Less than 3 months	4	(28.6%)	3	(30.0%)	7	(29.2%)
From 3 months through 1 year	3	(21.4%)	4	(40.0%)	7	(29.2%)
At least 1 year	5	(35.7%)	3	(30.0%)	8	(33.3%)
Never tested for HIV	2	(14.3%)	0	(0%)	2	(8.3%)
Initiated PrEP[f]	4	(28.6%)	N/A			
Initiated ART[e]	N/A		10	(100%)		

ART, antiretroviral therapy; N/A, not accessible; PrEP, pre-exposure prophylaxis; TGW, transgender woman.

a. Median, interquartile range; b. 2 missing values; c. Participants with male sex assigned at birth who reported a TGW or female gender identify; d. “Transwoman” (n=1) and “Female sex worker” (n=2); e. Excluding 19 known positive partners; f. 1 missing value.
